# Metabolic cost and mechanics of walking in women with fibromyalgia syndrome

**DOI:** 10.1186/1756-0500-6-420

**Published:** 2013-10-18

**Authors:** Renée S MacPhee, Kristen McFall, Stephen D Perry, Peter M Tiidus

**Affiliations:** 1 Department of Kinesiology and Physical Education, Wilfrid Laurier University, 75 University Ave W., Waterloo, ON N2L 3C5, Canada

**Keywords:** Fibromyalgia, Gait, Metabolic cost of walking, Pain

## Abstract

**Background:**

Fibromyalgia syndrome (FS) is characterized by the presence of widespread pain, fatigue, muscle weakness and reduced work capacity. Previous research has demonstrated that women with fibromyalgia have altered walking (gait) patterns, which may be a consequence of muscular pain. This altered gait is characterized by greater reliance on hip flexors rather than ankle plantar flexors and resembles gait patterns seen in normal individuals walking at higher speeds, suggesting that gait of individuals with fibromyalgia may be less efficient.

This study compared rates of energy expenditure of 6 females with FS relative to 6 normal, age and weight matched controls, at various walking speeds on a motorized treadmill. Metabolic measurements including V0_2_ (ml/kg/min), respirations, heart rate and calculated energy expenditures as well as the Borg Scale of Perceived Exertion scale ratings were determined at baseline and for 10 min while walking at each of 2, 4 and 5 km/hour on 1% grade. Kinematic recordings of limb and body movements while treadmill walking and separate measurements of ground reaction forces while walking over ground were also determined. In addition, all subjects completed the RAND 36-Item Health Survey (1.0).

**Findings:**

Gait analysis results were similar to previous reports of altered gait patterns in FS females. Despite noticeable differences in gait patterns, no significant differences (p > 0.05) existed between the FS and control subjects on any metabolic measures at any walking speed. Total number of steps taken was also similar between groups. Ratings on the Borg Scale of Perceived Exertion, the RAND and self-reported levels of pain indicated significantly greater (p < 0.05) perceived effort and pain in FS subjects relative to control subjects during walking and daily activities.

**Conclusions:**

The altered gait patterns and greater perceptions of effort and pain did not significantly increase the metabolic costs of walking in women with FS and hence, increased sensations of fatigue in FS women may not be related to alteration in metabolic cost of ambulation.

## Findings

According to the Centers for Disease Control and Prevention [1], fibromyalgia syndrome (FS) is characterized by the presence of “widespread pain, abnormal pain processing, sleep disturbance, fatigue, and often psychological distress”. Fibromyalgia is a somatic syndrome, which unfortunately lacks objective physical features and pathologic findings. Previous diagnostic criteria for fibromyalgia included, “tenderness at 11 or more of the 18 specific tender point sites” [2], however, recent revisions to the criteria due to growing concerns about the limited and correct use of tender point identification during physical examination has resulted in this diagnostic criterion being removed from the American College of Rheumatology’s diagnostic list [3]. Approximately 2-4% of the population may have fibromyalgia, it is most common in women, and generally affects individuals who are middle aged [4].

Several studies have noted significant differences in gait mechanics and walking strategies in individuals with fibromyalgia syndrome relative to a normal population.

Pierrynowski et al. [5] found that women with FS had altered gait patterns, which they may have adopted as a result of their muscular pain. Gait analysis, from lower extremity joint kinematics combined with measuring ground reaction forces while walking at self selected slow and fast speeds that were similar between groups, suggested that the gait of individuals with fibromyalgia may be less efficient than that seen in normal populations walking at normal speeds. The gait of individuals with fibromyalgia walking at normal speeds preferentially relied on hip flexors over ankle plantarflexors during gait and resembled gait patterns seen in normal individuals walking at higher rates of speed.

Auvinet et al. [6], conducted a gait analysis study with 14 FS subjects and 14 matched controls. FS walking speed, cycle frequency, stride length, side-to-side symmetry and step regularity was diminished or more poorly controlled than in matched controls. Overall, FS subjects were found to walk more slowly, which in turn decreased stride length and the cycle frequency at self selected preferred walking speeds.

Using the GAITRite™ system Jiménez et al. [7] reported that FS subjects demonstrated a significantly higher double support phase during the walking trials, reduced walking speed, decreased stride length, swing phase and single support time. Suggesting impaired gait parameters perhaps due to factors such as decreased levels of physical activity, increased body weight, fatigue and pain.

Dal et al. [8] reported similar oxygen cost and consumption for both the FS and control subjects during walking at self selected walking speeds. This may have been due to the slower self selected walking speeds adopted by the FS subjects relative to matched controls. Dal et al. suggested that the FS subjects were compensating for the pathology in their gait and that the Borg Scale of Perceived Exertion measures were higher in the FS subjects relative to controls despite slower walking speeds [8].

No study has yet compared the metabolic cost of walking in FS individuals with matched controls at similar walking speeds to determine if walking efficiency is influenced by the abnormal gait patterns exhibited in FS individuals and whether a potential increase in the energy cost of walking could therefore account for some of the fatigue reported in FS subjects.

Hence, the objective of this current study was to quantify energy expenditure and gait mechanics of females with FS relative to normal, age and weight matched controls, at matched slow, normal and fast walking speeds. It was hypothesized that altered walking patterns in FS individuals are metabolically more costly than those of normal individuals walking at matched speeds. If so, their higher metabolic costs and walking inefficiencies may partially explain the greater sensations of fatigue experienced by individuals with fibromyalgia when walking and performing other simple tasks. These findings could establish a quantifiable baseline for further research assessing treatment alternatives for individuals with fibromyalgia that would: a) attempt to improve their gait efficiency and reduce their energy expenditure while walking; and b) provide a quantifiable measure to determine the degree of gait impairment in individuals with fibromyalgia and the effectiveness of any treatment modality in altering their functional and walking abilities.

The Human Research Ethics Board at Wilfrid Laurier University provided approval to conduct the study, and informed consent was obtained from the subjects before participating in the study. Each subject reported to the laboratory without having exercised in the previous 24-hours, and at least four hours following her last meal. All subjects were required to wear flat-soled sport shoes throughout the study.

### Participants

Females with a confirmed diagnosis of Fibromyalgia Syndrome (FS) based on criteria established by the American College of Rheumatology were recruited for the study utilizing several strategies, including advertisements in two local newspapers, an email advertisement through a local fibromyalgia support group, and letters of information to rheumatologists practicing within the Region of Waterloo. The result was the recruitment of eight subjects. A convenience sampling method was used to recruit eight control subjects who were matched on age (+/-5 years), height (+/-10 cm) and weight (+/-4 kg) to the FS subjects. Subjects who had a previous or current injury, impairment and/or deformity of the lower limb(s) that could impede gait pattern and ability to walk on a motorized treadmill were excluded from the study. This resulted in the elimination of two subjects with FS (and their matched controls); one subject had a recent ankle injury, and one subject was scheduled for knee replacement surgery. The final sample consisted of six subjects with FS and six matched controls (Table 1). No significant differences (p > 0.05) in age, height or body weight existed between fibromyalgia and control groups. Prior to commencing the study, all subjects completed a brief medical history questionnaire, the Physical Activity Readiness Questionnaire (PAR-Q) [9], and the RAND 36-Item Health Survey (1.0) [10].

**Table 1:** Subject demographics

		**Fibromyalgia subjects**	**Control subjects**
		**n = 6**	**n = 6**
Age (years)	Mean (SD)	47.3 (10.4)	46.2 (9.1)
	(Min, Max)	(33,57)	(35, 57)
Height (cm)	Mean (SD)	168 (8.9)	167 (9.1)
	(Min, Max)	(160, 183)	(155, 180)
Weight (kg)	Mean (SD)	(79.1 21,8)	78.9 (19,5)
	(Min, Max)	(54.3, 107.5)	(57.9, 110.9 )
Length of illness (years)	Mean (SD)	10.8 (3.3)	n/a
	(Min, Max)	(6, 15)	

### Non-metabolic measurements

#### RAND 36-item health survey

The RAND 36-Item Health Survey (1.0)^5^ is an instrument that is based on the Medical Outcomes Study. The RAND, which consists of 36 closed-ended questions, measures eight dimensions of health: physical functioning (10 items); bodily pain (2 items); role limitations due to physical health problems (4 items); role limitations due to personal or financial problems (3 items); emotional well-being (5 items); social functioning (2 items); energy / fatigue (4 items); and, self-perceptions of health (5 items); change in health (1 item). The test items for each dimension are scored and averaged together in order to create scale scores that can range from 0 to 100. Higher scores represent a higher level of health function. The survey was completed by each subject prior to commencing her walking trials on the treadmill.

#### Self reported level of pain and level of exertion

Using a numerical pain scale (ranging from 0 = no pain, to 10 = worst pain imaginable), subjects were asked to self-report the level of pain they experienced on their best and worst days, as well at baseline and while walking on the treadmill. Using the Borg Scale of Perceived Exertion [11] subjects were also asked to rate the perceived level of exertion they experienced in walking on the treadmill. Pain and exertion data was captured at baseline and again at minutes 3 and 8 during each of the three 10-minute walking periods.

#### Gait analyses / force plate

Gait mechanics and muscle power patterns were determined by evaluation of kinematic data (OptoTrak 3020, Northern Digital, Inc, Waterloo, ON, CAN) and ground reaction forces. Infra-red markers were placed on the left lateral side of individuals positioned at the joints of interest (e.g., fifth metatarsal, heel, ankle, knee, hip, shoulder, elbow, wrist, and temple), to record each joint’s angular motion during the treadmill walking. Additional walking trials across a force platform (Advanced Mechanical Technology Inc., Watertown, MA, USA) were recorded in tandem with kinematic data at similar speeds (within ± 5%) to those of the treadmill walking to provide force and kinematic data for the calculation of inverse dynamics to yield individual joint movement, work, and power profiles, as well as work done on the body as a whole during the walking cycle. These measurements provided insight as to where the inefficiencies might be occurring. The techniques have been used in various previous studies involving older female populations [12].

### Metabolic measurements

Direct measure of breath-by-breath oxygen consumption during rest and at the three walking speeds was assessed using a computerized Cosmed portable K4b2 metabolic system. Rate of oxygen uptake at rest and at 3 and 8 min of walking at each speed was determined and expressed as ml O2 consumed per kg body weight per minute. Aerobic energy expenditure expressed as kilocalories per minute (Kcal/min) was also calculated at these time points from expired gases using the formula, “3.781 × VO_2_ + 1.237 × VCO_2_” as assessed by the Cosmed metabolic system and based on the recommendations of Elia and Livesey [13]. Energy expenditure was also determined per Kg BW. The K4b2 unit was calibrated prior to use on each subject.

Upon completion of the on ground walking trials, a latex free facemask, which covered the nose and mouth, was then applied to the subject. The facemask, which had ventilation holes, allowed the subject to breathe normally. The facemask was held in place with a headband and was secured with Velco straps. The facemask was connected directly to the K4b2 portable unit, which was worn in a harness style on the upper back and chest area and captured each expiratory breath. All breath-by-breath data were sent to a computer via telemetry. A heart rate monitor was also applied around the upper torso, slightly below the sternum. Baseline metabolic measurements were made for 10 minutes on subjects while they were seated quietly.

At the end of the baseline period, the markers that were used during the initial gait analyses were checked to ensure that correct placement had been maintained; subjects were then placed on a motor driven treadmill. Breath-by-breath analyses continued while subjects walked for ten minutes on the treadmill at a 1% grade at each of 2 km/hour (slow walking), 4 km/hour (normal walking), and 5 km/hour (fast walking). The markers captured the subjects’ gait position at minutes 3 and 8 during each of the ten-minute walking periods. For safety purposes each subject was equipped with an emergency release device, which was attached to both the subject and the treadmill. If the connection between the emergency release device and the subject was broken, the treadmill was programmed to automatically stop. Because the first FS subject was unable to walk on the treadmill without holding onto at least one handrail due to a fear of falling, all remaining subjects (FS and control) were instructed to keep their right hand on the right handle of the treadmill throughout the testing period in order to maintain a consistent and controlled trial.

### Participants

#### FS Subjects

With respect to the medical history of the subjects with FS, one subject reported that she suffered from hypertension, one subject reported a history of migraine headaches, and one other subject reported that she suffered from asthma. Three subjects indicated that they had been previously diagnosed with either osteopenia, osteoarthritis, or arthritis. Three subjects reported a remote history of hip, knee, or ankle injuries, which at the time of study did not impair their ability to ambulate. None of the subjects reported a history heart disease, diabetes, stroke, or prosthetic limbs. When asked if they were taking any medications (including prescription, over the counter, natural, homeopathic, and herbal remedies), all subjects responded in the affirmative. Prescriptions medications that were noted as being taken in the FS group were from the following pharmaceutical classes: analgesic (non-narcotic); analgesic (narcotic); antidepressant; antiemetic; anti-estrogen; anti-inflammatory; anxiolytic; bronchodilator; sedative / hypnotic; and, thyroid hormone. Of note, is that only one subject reported taking prescription narcotic analgesics for pain associated with her FS; the remaining subjects reported using products that did not contain narcotics (e.g., Extra-Strength Tylenol™, Advil™, Ibuprofen™). Excluding over-the-counter analgesics, the most frequently reported non-prescription medications were vitamins and folic acid. All subjects were non-smokers. Two subjects reported that they were physically active on a daily basis, whereas the remaining subjects indicated they did not participate in physical activity at all.

#### Matched control subjects

Two of the control subjects reported they suffered from asthma, one subject reported she had both heart disease and hypertension, and three subjects reported they suffered from migraine headaches. None of the control subjects had a history of diabetes, stroke, prosthetic limbs, skeletal disease (e.g., osteopenia) or rheumatoid condition (e.g., arthritis, osteoarthritis). Three subjects reported a remote history of knee and/or ankle injuries, which at the time of study did not impair their ability to ambulate. The most commonly prescribed medications included anti-depressants, anti-hypertensives, antilipid, and bronchodilators. The most commonly reported over-the-counter (non-prescription) medications included analgesics and vitamins. Three subjects reported that they were smokers. Two subjects reported being physically active one to two times per week, while the remaining four subjects reported that they did not participate in physical activity.

### Data analyses

Univariate statistical procedures were used to provide descriptive overviews of both the non-metabolic measures (i.e., RAND, self-reported levels of pain, self-reported levels of exertion, and number of steps taken while walking on the treadmill) and metabolic measures of interest (i.e., V0_2_, V0_2_/kg, respirations, heart rate, EEm, EEbsa, EEkg, and METS). In light of the small sample size, analyses at the bivariate level were completed using non-parametric statistical tests (Wilcoxon Rank Sum Test) [14] in order to identify significant differences between the two groups of subjects. To calculate joint powers, ground reaction forces were recorded in conjunction with kinematics during the over-ground walking trials. The joint powers were calculated using procedures outline in Winter [15].

### Non-metabolic measurements

#### RAND 36-item health survey (Version 1.0)

With the exception of three dimensions (e.g., role limitations due to emotional problems, emotional well being, and, general health), significant (P < 0.01) differences between the two groups of subjects were noted on the remaining five dimensions, pain, physical health, energy/fatigue, physical functioning and role limitations due to physical health. In each of dimensions the FS group scores were significantly higher than the controls indicating greater fatigue, pain and functional limitations.

#### Self-report measurement of pain

Using a numerical pain scale of 0-10, where 0 was equated with 'no pain’ and 10 was the 'worst pain imaginable’, subjects were asked the following two questions: a) “On days when you are feeling your best, how would you rate your pain?”, and, b)”On days when you are feeling your worst, how would you rate your pain?” On both pain dimensions the FS subjects scored significantly (P < 0.01) higher than the controls (mean ± SD.fibromyaliga vs control: best days 3.1 ± 1.5 vs 0.0 ± 0.4, worst days 9.5 ± 0.8 vs 3.0 ± 2.9). Using the same numerical rating scale, subjects were then asked to report their level of pain at minutes 3 and 8 during baseline and at each of the three 10-minute walking periods on the treadmill. Again the FS subjects reported significantly (P < 0.01) higher pain at each of the walking time points. The fibromyalgia subject’s pain scores progressed from means of 3.8 to 5.75 as the walking speeds increased while the control subjects reported pain (0.0) at any of the walking speeds.

#### Self-reported level of exertion

At various time points throughout the study, the Borg Scale of Perceived Exertion was used to have the subjects rate their perceived levels of exertion between 0 (nothing at all) and 10 (very, very hard). Subjects were asked to report their level of exertion at minutes 3 and 8 during each of the three 10-minute walking periods on the treadmill. At each of the walking speeds the FS subjects reported significantly (P < 0.01) greater perceived exertion than the control subjects progressing from means of 3.0 to 5.8 as walking speed and time increased while the control subjects reported perceived exertion progressing from means of 1.0 to 2.0 as walking speed and time increased.

#### Gait analyses: total steps taken

The following table summarizes the data related to the total number of steps that each subject took during the walking trial on the treadmill. There was no significant difference between the two groups on this measure (P > 0.05).

#### Gait analyses: hip and ankle power

**Figure 1:**
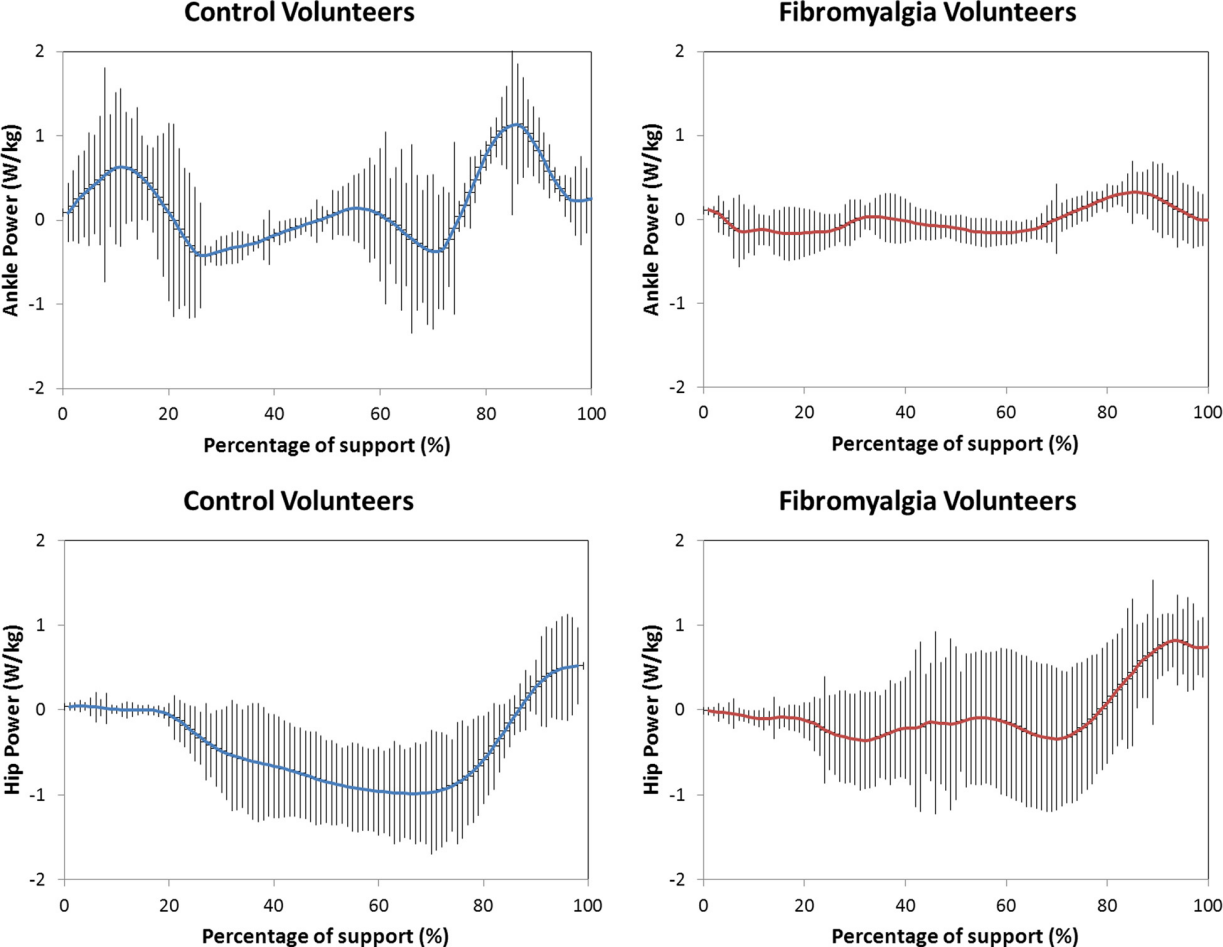
Representative hip and ankle power for repeated trails (vertical lines represent standard error) of control and fibromyalgia participants.

Table 2 provides a summary of the hip and ankle power for the FS and control subjects. Figure 1 depicts representative ankle and hip power for repeated trials of a FS and control subject.

**Table 2:** Comparison of hip power to ankle power during the end of the stance phase

	**Fibro (n =5)**	**Control (n = 2)**
Hip power (W/kg)	1.146 (0.379)	0.768 (0.658)
Ankle power (W/kg)	0.639 (0.589)	1.325 (0.399)
Ankle/hip power ratio	0.558	1.725

Data were scaled and normalized.

Note: Due to technical issues joint powers in several of the control participants were not calculated.

### Metabolic measurements

Tables 3 and 4 provides a summary of the mean (SD), and range (minimum, maximum) for the values of V0_2_ /kg (ml/min/kg) and calculated energy expenditure (Kcal/min) respectively, which were captured at the 3 and 8 minute time points at baseline and while subjects walked for ten minutes on the treadmill at a 1% grade at each of 2 km/hour (slow walking), 4 km/hour (normal walking), and 5 km/hour (fast walking). Respiration, heart rate, and calculated energy expenditure in metabolic equivalents (METS) were also determined but exhibited no significant differences (P > 0.05) between the two groups of subjects (data not shown). Despite noticeable differences in gait patterns, no significant differences (P > 0.05) existed between the fibromyalgia subjects and the control subjects in oxygen consumption (ml/kg/min) or calculated energy expenditure (Kcal/min) at either of the 3 or 8 min time points at any of the walking speeds. Energy expenditure while walking expressed as Kcal/min.kg BW (data not shown) also did not result in any significant (P > 0.05) differences between groups at any of the time points or walking speeds.

**Table 3:** **V0**_**2**_**/kg (ml/min/kg)**

**Subject type**	**Baseline**	**Baseline**	**2 km**	**2 km**	**4 km**	**4 km**	**5 km**	**5 km**
	**(3 min.)**	**(8 min)**	**(3 min.)**	**(8 min.)**	**(3 min.)**	**(8 min.)**	**(3 min.)**	**(8 min.)**
	**Mean (SD)**	**Mean (SD)**	**Mean (SD)**	**Mean (SD)**	**Mean (SD)**	**Mean (SD)**	**Mean (SD)**	**Mean (SD)**
	**(Min, Max)**	**(Min, Max)**	**(Min, Max)**	**(Min, Max)**	**(Min, Max)**	**(Min, Max)**	**(Min, Max)**	**(Min, Max)**
FS	3.2	3.9	6.7	8.3	10.0	11.0	11.8	12.2
	(1.1)	(2.7)	(3.7)	(3.1)	(3.4)	(3.0)	(2.3)	(5.6)
	(1.93,4.75)	(1.3,8.5)	(1.4,12.6)	(5.2,13.3)	(7.4,16.4)	(7.8,15.5)	(9.8,13.3)	(9.2,16.5)
Controls	2.71	2.6	7.3	7.0	9.1	9.1	11.3	11.7
	(0.8)	(0.8)	(2.1)	(1.9)	(1.8)	(1.5)	(2.4)	(1.6)
	(1.7,4.0)	(2.1,4.1)	(5.2,10.8)	(5.1,9.2)	(6.711.1)	(7.2,11.1)	(8.7,12.6)	(10.0,14.7)
Wilcoxon rank sum test	p = n.s	p = n.s.	p = n.s.	p = n.s.	p = n.s.	p = n.s.	p = n.s.	p = n.s.
	(p > .29)	(p > .34)	(p > .41)	(p > .30)	(p > .47)	(p > .12)	(p > .35)	(p > .41)

**Table 4:** EEm (Kcal/min)

**Subject type**	**Baseline**	**Baseline**	**2 km**	**2 km**	**4 km**	**4 km**	**5 km**	**5 km**
	**(3 min.)**	**(8 min)**	**(3 min.)**	**(8 min.)**	**(3 min.)**	**(8 min.)**	**(3 min.)**	**(8 min.)**
	**Mean (SD)**	**Mean (SD)**	**Mean (SD)**	**Mean (SD)**	**Mean (SD)**	**Mean (SD)**	**Mean (SD)**	**Mean (SD)**
	**(Min, Max)**	**(Min, Max)**	**(Min, Max)**	**(Min, Max)**	**(Min, Max)**	**(Min, Max)**	**(Min, Max)**	**(Min, Max)**
FS	1.17	1.5	2.45	3.1	3.7	4.1	4.5	4.4
	(0.4)	(1.2)	(1.19)	(1.0)	(0.9)	(1.1)	(1.0)	(1.9)
	(0.9, 1.72)	(0.5, 3.8)	(0.5, 3.8)	(1.9, 4.8)	(2.6, 4.9)	(2.8, 6.1)	(3.4 6.1)	(3.4, 5.0)
Controls	1	1.0	2.8	2.7	3.5	3.6	4.4	4.6
	(0.3)	(0.4)	(0.9)	(0.8)	(1.0)	(101)	(1.3)	(1.3)
	(0.7, 1.5)	(0.5, 1.2)	(1.5, 3.6)	(1.6, 3.4)	(2.3, 4.3)	(2.2 4.9)	(2.6, 5.8)	(3.0, 6.2)
Wilcoxon rank sum test	p = n.s.	p = n.s.	p = n.s.	p = n.s.	p = n.s.	p = n.s.	p = n.s.	p = n.s.
	(p > .19)	(p > .35)	(p > .35)	(p > .41)	(p > .40)	(p > .35)	(p > .53)	(p > .24)

## Availability of supporting data

No significant differences in oxygen consumption or calculated energy costs were seen between female FS subjects and age and weight matched controls at three different walking speeds despite significant differences in gait mechanics and higher levels of pain, perceived exertion and discomfort reported in FS subjects. It is possible that the differences in gait mechanics between our groups were not of sufficient magnitude to significantly alter energy expenditure and oxygen consumption measures between groups. Although technical difficulties limited the numbers of subjects whose gait could be completely analyzed, results from our study did suggest that the gait mechanics differed between groups in a manner similar to that previously reported by Pierrynowski et al. [5]. The joint power results in this study demonstrated the same increased reliance, in the fibromyalgia participants, on hip power in relationship to ankle power as found in a previous study by Pierrynowski et al. [5]. These differences did not result in significant differences in oxygen consumption or energy expenditure at matched slow or faster walking speeds. Although various other clinical populations have reported greater energy expenditure due to altered gait mechanics, such findings are not universal [16] and at least one other study has reported a lack of difference in energy expenditure at self selected walking speeds between FS and control subjects [8] Hence, altered gait mechanics in women with FS may not account for the increased fatigue expressed by the subjects with FS based on any significant differences in energy expenditure or higher levels of oxygen consumption. Therefore increased daily fatigue in women with FS relative to controls may not be related to differences in energy expenditure while walking. Alternatively, although not measured in this study, local muscle fatigue due to altered gait mechanics may have been greater in the FS subjects relative to controls, possibly contributing to fatigue and pain sensations. Given that there was no significant differences between the groups with respect to oxygen consumption or energy expenditure while walking it may be that, FS feel more “fatigued” in part because FS is accompanied by increased sensations of local and global muscle pain.

Several limitations in the study may have influenced our results. All subjects were unfamiliar with the walking surface; therefore all subjects were required to hold onto the treadmill handle with their right hand (so as not to interfere with the markers on the left side of the body where the biomarkers had been placed). This additional support may have had minor influences on the altered gait patterns, oxygen consumption and energy expenditure measures relative to free walking. Two of the FS subjects reported that they exercised on a regular basis. In light of the small sample size, their higher level of physical fitness may have skewed the metabolic measurements for the group of FS subjects. As noted by Water and Mulroy [16] increased cardiovascular fitness in clinical populations with altered gait patterns, may lead to an increased ability to tolerate higher rates of energy consumption in order to maintain a more normal walking speed. Additionally, regular exercise will result in enhanced well being and reduced muscle pain in FS subjects [17]. The time spent walking on the treadmill (at each speed and overall) was relatively short and as such, we might not have been able to readily capture metabolic changes that might have been manifested in longer walking trials that could have induced greater fatigue. The simplified marker setup may have introduced some minor error with regard to calculating the ankle and hip powers, however the results were very similar to the Pierrynowski et al. [5] study on a similar population. Additionally, a relatively small sample size may have possibly influenced the gait and energy expenditure results; however, strong agreement with earlier gait data was demonstrated and little difference was noted between groups in energy expenditure and oxygen consumption. Nevertheless, a wider sample size of FS subjects may have produced a greater variation in energy expenditure with possibly some subjects exhibiting greater differences from controls. Further research will be required with larger subject populations to further verify our initial findings.

Within these potential limitations of the study, we found that female FS subjects did not demonstrate significantly greater oxygen consumption or energy expenditure during treadmill walking at slower or faster speeds than age and weight matched normal controls despite significant differences in gait mechanics and greater discomfort and fatigue during walking or daily life. Hence, energy expenditure during walking may not explain differences in fatigue or discomfort experienced by FS individuals relative to normal controls.

## Competing interests

The authors have no competing financial, patent or non-financial interests in relation to this study.

## Authors’ contributions

RSM carried out the subject recruitment and experiment coordination, data collection, data analysis and participated in its design and the draft the manuscript. KM assisted with the data collection, experiment coordination and data analysis, SDP participated in its design, performed data analysis and helped draft the manuscript. PMT conceived the study, participated in its design and helped draft the manuscript. All authors have read and approved the manuscript.
